# Deep learning-based classification model for GPR151 activator activity prediction

**DOI:** 10.1186/s12859-023-05369-y

**Published:** 2023-06-09

**Authors:** Huangchao Xu, Baohua Zhang, Qian Liu

**Affiliations:** 1 Computer Network Information Center, Chinese Academy of Sciences, Dongsheng Sourth Street No.2, Haidian District, Beijing, 100190 China; 2 University of Chinese Academy of Sciences, No.1 Yanqihu East Rd, Huairou District, Beijing, 101408 China

**Keywords:** Activity prediction, Deep learning, Feature extractor

## Abstract

**Background:**

GPR151 is a kind of protein belonging to G protein-coupled receptor family that is closely associated with a variety of physiological and pathological processes.The potential use of GPR151 as a therapeutic target for the management of metabolic disorders has been demonstrated in several studies, highlighting the demand to explore its activators further. Activity prediction serves as a vital preliminary step in drug discovery, which is both costly and time-consuming. Thus, the development of reliable activity classification model has become an essential way in the process of drug discovery, aiming to enhance the efficiency of virtual screening.

**Results:**

We propose a learning-based method based on feature extractor and deep neural network to predict the activity of GPR151 activators. We first introduce a new molecular feature extraction algorithm which utilizes the idea of bag-of-words model in natural language to densify the sparse fingerprint vector. Mol2vec method is also used to extract diverse features. Then, we construct three classical feature selection algorithms and three types of deep learning model to enhance the representational capacity of molecules and predict activity label by five different classifiers. We conduct experiments using our own dataset of GPR151 activators. The results demonstrate high classification accuracy and stability, with the optimal model Mol2vec-CNN significantly improving performance across multiple classifiers. The svm classifier achieves the best accuracy of 0.92 and F1 score of 0.76 which indicates promising applications for our method in the field of activity prediction.

**Conclusion:**

The results suggest that the experimental design of this study is appropriate and well-conceived. The deep learning-based feature extraction algorithm established in this study outperforms traditional feature selection algorithm for activity prediction. The model developed can be effectively utilized in the pre-screening stage of drug virtual screening.

## Background

G protein-coupled receptors (GPCR) are the largest family of membrane protein receptors in the mammalian genome, widely distributed in the central nervous system, immune system, cardiovascular and other organs and tissues. They are involved in both physiological and pathological processes including nociception. In recent years, GPCR research is highly sought after by pharmaceutical companies due to the potential of GPCRs as candidate targets in the search for new therapeutics. GPR151 is an orphan receptor belonging to the class A GPCR family that is highly enriched in receptor nuclei neurons. It plays a role in regulating mood, stress, nicotine withdrawal and preventing obesity. GPR151 is abundantly expressed in the dorsal root ganglia and is closely associated with nociception, making it a potential drug target for treating a variety of psychiatric, neurological, and metabolic disorders. Xia et al. [1] first identified the molecular and cellular mechanisms through which GPR151 can modulate neuropathic pain by regulating P2X3 function and microglia activation. Jiang et al. [2] found that GPR151 acts as a $G\beta \gamma$-coupled receptor to induce ERK (signal-regulated kinase)-dependent neuroinflammation and may be a potential drug target for the treatment of trigeminal neuralgia. Beatriz et al. [3] demonstrated that GPR151 regulates sensitivity and aversion to nicotine, indicating that small molecule modulators of this receptor may be useful to treat nicotine addiction. The findings of Ewa et al. [4] showed that GPR151 can regulate gluconeogenesis in the liver, highlighting the therapeutic potential of targeting GPR151 for the treatment of metabolic diseases. The above studies indicate that investigations into activators targeting GPR151 is relevant and important.

Traditional screening of molecular activity is accomplished through high-throughput screening experiments. Existing techniques for investigating GPR151 activators are usually cell-based experimental studies which are expensive in research progress. Drug virtual screening technology has emerged as a cost-effective and efficient method for modern drug development, offering a new way to reduce costs and increase the probability of drug discovery. Despite the advantages of virtual screening methods, they still face certain challenges. Firstly, when the active pocket of a target protein is unknown or cannot be determined, it may be necessary to traverse the protein structure space to obtain optimal docking results, which can decrease the accuracy of docking results and lead to computational inefficiencies. Secondly, traditional molecular docking-based virtual screening can be time-consuming and labor-intensive. The resource and time consumption are still significant and unaffordable for many users even when using high-throughput screening to optimize performance.

**Fig. 1:**
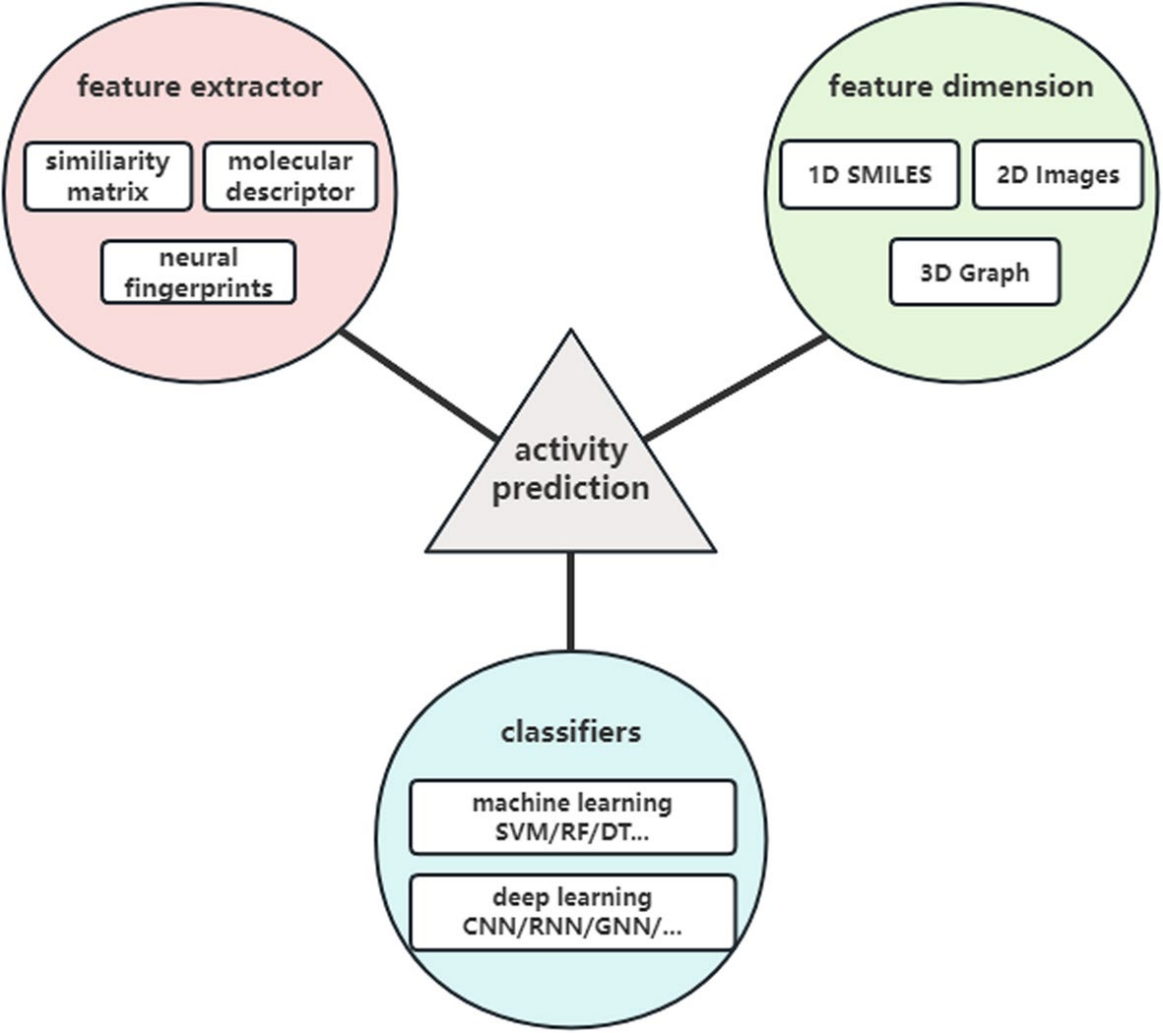
Classification of molecular activity prediction techniques based on artificial intelligence-assisted drug design

The PubChem database has incorporated four high-throughput screening experimental datasets for small molecule activators of GPR151 since 2020. The comprehensive dataset provides a valuable resource for conducting broader molecular activity studies of GPR151 activators. The recent surge in machine learning and deep learning techniques has accelerated the development of intelligent systems in the field of molecular research. Artificial intelligence-assisted drug design (AIDD) [5] can be used for molecular activity prediction. This approach is not only effective in reducing the time and cost associated with experimental screening, but also in expanding the chemical space that can be explored. Deep learning models have demonstrated remarkable proficiency in handling high-dimensional and complex features. Such methods are beneficial in reducing dependence on expert knowledge and improving the predictive capabilities of the models. Feature representation and model selection are key aspects of molecular deep learning [6, 7]. Current AIDD-based activity prediction methods can be broadly categorized based on feature dimensions, extraction methods and classifiers, as illustrated in Fig. 1.

Molecular features can be SMILES sequences [8], molecular images [9] or three-dimensional structures and coordinates, with the development of graph neural networks [10, 11] in recent years. Molecular feature extraction methods are normally divided into molecular descriptors, similarity matrix and neural fingerprints. Molecular fingerprint is a form of qualitative descriptor, which represents molecular structure and substructure through data encoding. RDKit fingerprints [12], MACCS Keys [13], ECFPs [14] and Daylight fingerprints [15] are widely used. Similarity matrix-based methods typically employ molecular sequences or molecular descriptors to calculate sequence similarity matrix for proteins and compounds. Another approach known as neural fingerprint was proposed by Merkwirth et al. [16], which mapped discrete chemical structures of compounds to a continuous vector space using deep neural networks. It has emerged as a popular method for molecular activity prediction, with commonly-used techniques including AttentiveFP [17], NeuralFP [18] and FP-GNN [19]. Logistic regression, K-nearest neighbor, random forest, decision tree and support vector machine [20] are all traditional classifiers with well-established libraries, fast training speeds, and wide applicability in molecular activity research. Meanwhile, deep learning models such as convolutional neural networks (CNN) [21], long and short-term memory neural networks (LSTM) [22] and generative adversarial networks (GAN) [23] are increasingly favored in the field. Among the studies related to drug activity prediction, representative unsupervised learning models include Mol2vec [24], which utilizes the Word2vec [25] model to learn vector representations of molecular substructures. By summing the vectors of individual substructures, compounds can ultimately be encoded as vectors. Mol2vec is a useful library for molecular feature extraction.

This paper proposes a data collection method aimed at assisting molecular docking computations, thereby facilitating the rapid virtual screening of large molecular databases for drug discovery. The method incorporates molecular docking computation and high-throughput experimental data to generate consistent GPR151 activator datasets. Additionally, we propose an improved algorithm which utilizes the idea of bag-of-words model in natural language processing to densify the sparse fingerprint vector. We also systematically compare the performance of various classical feature selection algorithms, deep learning models and traditional classifiers for molecule activity prediction tasks and find out the best model Mol2vec-CNN. To assess generalization performance, an experiment based on ZINC sub database is performed for well-performing deep learning models. The activity prediction labels of the trained model show an agreement of over 70% with results obtained from molecular docking software. These findings have a significant impact on the efficiency of screening active compounds from large molecule databases.

## Methods

### Molecular feature extraction

A molecular fingerprint is a condensed representation of a molecule that encodes its structural features into fixed-size arrays of bits for comparison. The typical process is to extract the molecular fragments and then hash them to generate bit variables, where each bit relates to a molecular fragment. This study employs two molecular fingerprint algorithms: topological fingerprint (RDKFP) and morgan fingerprint (MorganFP). RDKit version 2022.3.5 [26] is applied to compute fingerprint features, with the topological fingerprint parameters set as default values and morgan fingerprint using radius=2 and nBits=2048 to obtain feature lengths of 2048. Mol2vec [24] is an unsupervised machine learning model inspired by natural language processing techniques, which learns vector representations of molecular substructures with similar chemical structures. Mol2vec encodes compounds into vectors by summing individual substructure vectors, overcoming issues such as sparsity and bit conflicts commonly associated with feature representations. This provides a robust foundation for constructing molecular activity prediction models using supervised learning.

In this paper, we use traditional feature selection methods and deep learning methods to carry out further extraction for molecular fingerprint and Mol2vec to enhance model characterization capability. The study employs three traditional feature selection methods, namely principal component analysis (PCA) [27] linear discriminant analysis (LDA) [28] and decision tree algorithm (DTA) [29], as well as common deep neural network structures, namely CNN [21], LSTM [22] and bidirectional long short-term memory (Bi-LSTM) [30]. CNNs are feed forward neural networks including convolutional computation, which are outstanding in computer vision field. In drug activity prediction research, one-dimensional molecular sequences processed by molecular fingerprint and other feature extraction methods can serve as input to 1D-CNN. LSTM is a special type of recurrent neural network (RNN) to overcome gradient explosion or disappearance in the original RNN when processing longer sequence data. Bi-LSTM is a variant of the LSTM structure, consisting of two LSTMs superimposed on top and bottom together, with output jointly determined by the states of both LSTMs. To address the bit-sparse characteristics of original features extracted from molecular fingerprints, a preprocessing algorithm inspired by the bag-of-words model is proposed. Specifically, each bit in the fingerprint vector is treated as a vocabulary, with the *nth* feature corresponding to code $n+1$ ($0\le n \le 2047$, *n* is an integer). Each compound is seen as a sentence and the bit marked as 1 in the molecular fingerprint indicates the presence of the word in the sentence, with the corresponding code recorded. All valid bit numbers in the 2048-dimensional fingerprint are traversed to acquire a coding vector, then the code matrix is padded and input into the embedding layer of the neural network to transform the number matrix into dense feature vector. The Mol2vec features (100-dimensional) are processed using the open library (https://github.com/samoturk/mol2vec) and fed into different deep models for further feature extraction.

**Fig. 2:**
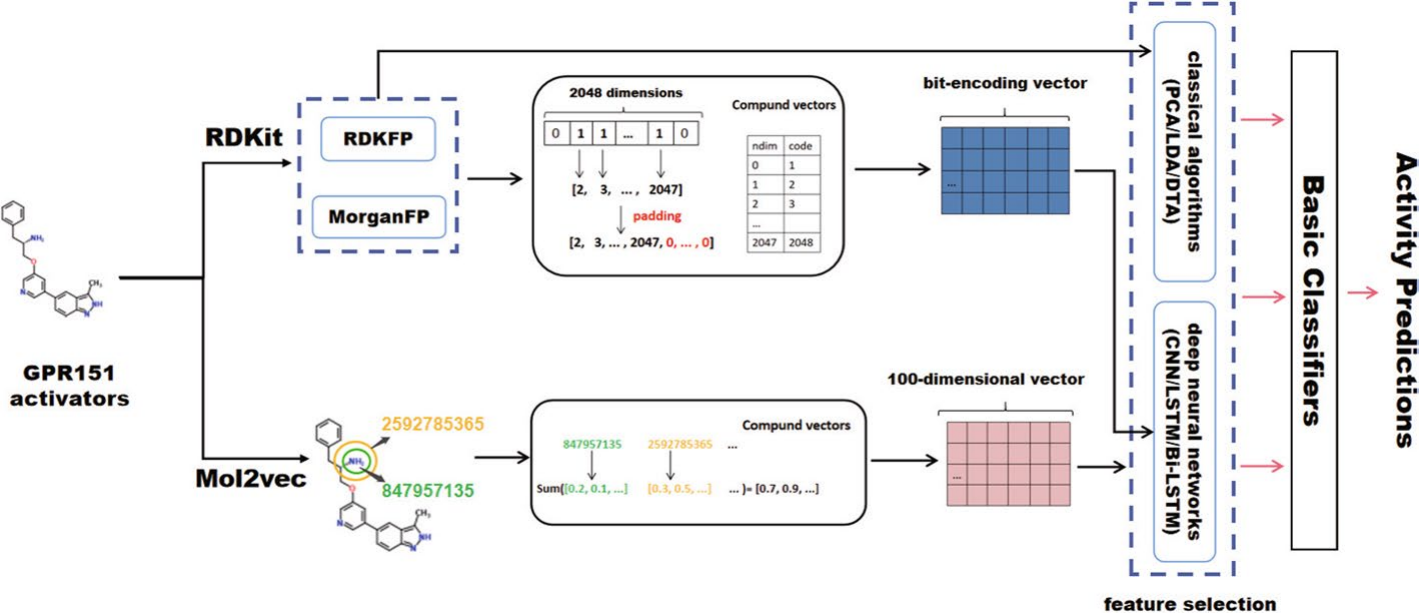
The flowchart of GPR151 activators activity prediction pipeline. The pipeline contains two types of molecular features, deep neural networks and traditional feature selection algorithms for comparison, followed by different classifiers to predict activator activity labels

### Classification

After using traditional algorithms and deep neural network for molecular feature extraction, this study employs five basic machine learning classifiers, namely logistic regression (LR), k-nearest neighbor (KNN), random forest (RF), decision tree (DT), and support vector machine (SVM) for activity classification. These classifiers are also compared with the softmax classifier of the deep neural network. The key flow is illustrated in Fig. 2.

## Results

### Docking preparation

The available conformation of the GPR151 receptor has an influence on molecular docking results, with the receptor’s activation state also playing an important role [31]. Previous studies demonstrate significant activation of GPR151 under acidic conditions, with maximum activation observed at pH 5.8. This leads to an increase in the receptor’s binding ability to ligands [32]. Dueto the lack of crystal structure and endogenous ligand for GPR151, this study utilizes AlphaFold2 [33] to predict the receptor’s three-dimensional structure, followed by molecular dynamics simulations to relax the structure and obtain a reasonable starting point for molecular docking. The simulations are conducted using the GROMACS 2020 software [34], with the residue protonation state set at pH 5.8, the AMBER ff14B force field and the SPC solventization model. A cubic box is utilized and Na and Cl equilibrium ions are added. The structures are energy minimized using the steepest descent method and subjected to molecular dynamics simulations for 10 ns at NPT ensemble in 2*fs* steps, following equilibration at NVT and NPT ensemble for adequate sampling through energy fluctuations, Root-Mean-Square-Deviation (RMSD) and Root-Mean-Square-Fluctuation (RMSF). The modeled GPR151 receptor is found to have an average energy of $-4.3e+06$ KJ/mol, with an energy fluctuation of less than 3%. For the conservative helical region, the RMSD averaged 0.3 nm with RMSF less than 0.5 nm, indicating small residue fluctuations. In contrast, the LOOP region exhibits greater structural flexibility in the simulations, leading to large RMSD and RMSF values. However, as the LOOP region is not involved in ligand docking, the simulations generates a reasonable three-dimensional structure.

After obtaining a reasonably relaxed three-dimensional structure, a large active pocket is selected based on the common structural characteristics of the seven helices of GPCRs.The helical region is enclosed and molecular docking calculations are performed using AutoDock Vina [35] and AutoDock GPU [36] software to exclude the influence of different docking algorithms. AutoDock Vina employs a gradient-based Iterated Local Search (ILS) search algorithm and an empirical-based scoring function, whereas AutoDock GPU utilizes Lamarckian Genetic Algorithm (LGA) [37] global conformational search combined with Solis-Wet structural search algorithm and a force field-based scoring function. Ten independent searches are conducted for each docking and the binding energy is calculated. The structure with the lowest binding energy is selected and combined with experimental results to determine the binding activity threshold $\alpha$.

The hardware environment for molecular dynamics and molecular docking calculations is the “ORISE” supercomputer with a single node equipped with 32 core x86 processors and 4 GPU accelerators at a base frequency of 2.0 GHz. AutoDock Vina calculations are performed on 32 CPU cores with 8 cores running in parallel intranode. AutoDock GPU utilizes 4 GPU cards to perform 4 tasks simultaneously intranode.

### Data collection and splitting

The GPR151 activator molecular datasets in this paper are gathered from PubChem Bioassay (https://pubchem.ncbi.nlm.nih.gov/bioassay/), as shown in Table 1. All four datasets are provided by the Scripps Research Institute Molecular Screening Center and the receptor protein is G protein-coupled receptor 151. The other three datasets were generated through high-throughput screening experiments, while activity in AID 1508610 was determined via high-throughput reaction experiments in the year of 2020.

**Table 1:** List of GPR151 activator datasets in PubChem Bioassay

S. No.	BioAssay AID	Total No. of compounds	Active compounds	Inactive compounds	BioAssay type
1	1508602	646675	6756	639919	Screening
2	1508608	2275	18	2257	Screening
3	1508609	6747	6	6741	Screening
4	1508610	600	83	517	Confirmatory

This study focuses on two datasets: AID1508602 and AID1508610, containing a significant number of active molecules for experimentation. As the model is intended to facilitate molecular docking and reduce the imbalance between active and inactive classes, a consistent data collection process is implemented for AID1508602. Docking experiments are conducted on the activators and GPR151 receptor protein within AID1508602, following experiment settings outlined in section docking preparation. The binding energies are subsequently compared with the activity labels in the original dataset.We initially set a threshold of $\alpha$ for binding energy, whereby binding energies less than $\alpha$ were considered active, while those greater than $\alpha$ were categorized as inactive. The threshold $\alpha$ is established as $-$8.6 kcal/mol based on an 80% consistency between the computational and experimental results obtained from Autodock Vina [35] and AutoDock GPU [36]. Based on this threshold, molecules with a binding energy lower than $-$8.6 kcal/mol are considered active, while those with a binding energy greater than $-$8.6 kcal/mol are classified as inactive.

After acquiring consistent results, we select 6066 molecules from the AID1508602, out of which 1066 are active. In the case of the AID1508610, all records are included in the final dataset, considering its small size and moderate ratio of two type molecules. This is done to increase noise and enhance the model’s robustness. The final GPR151 activator dataset is presented in Table 2, with test sets divided at the ratio of 0.2, as indicated in Table 3.

**Table 2:** The detail of GPR151 activator dataset constructed in this paper

Source BioAssay	Active	Inactive	Total
AID 1508602	1066	5000	6066
AID 1508610	83	517	600
Total	1149	5517	6666

**Table 3:** The train and test set splitting method for GPR151 activator dataset

GPR151	Active	Inactive	AID1508602	AID1508610	Total
Train	906	4426	4847	485	5332
Test	243	1091	1219	115	1334

### Model parameters

**Fig. 3:**
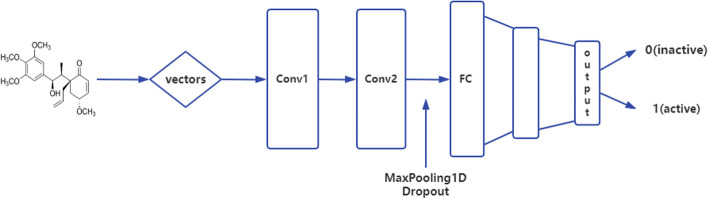
The CNN network architecture used in this paper

In this study, TensorFlow and Keras deep learning libraries are employed to train CNN, LSTM, and Bi-LSTM models, with backpropagation used to optimize the weights between hidden layers. The CNN network architecture designed for molecular fingerprint and Mol2vec features is illustrated in Fig. 3, comprising two convolutional layers (with filters of 32 and 16, kernel size of 8, respectively), a pooling layer (with pool size of 3) and multiple dense layers. The relu activation function is employed in all layers except for the last one. The category probabilities are output through the sigmoid activation function. The LSTM and Bi-LSTM models’ one-way loop structure is set to 128 and 64 when the input features are molecular fingerprints and Mol2vec. The maximum number of layers is set to two. The model optimizer is Adam, with the learning rate 0.00025. We test multiple learning rates from the parameter list [0.0001, 0.00025, 0.0005, 0.001] to select the most effective one. Binary cross-entropy is selected as the loss function and the accuracy is applied to evaluate the model’s performance. The number of iterations (epoch) and batch size are adjusted based on the different molecular features and network structures. For instance, Mol2vec-CNN model has an setting of epochs=100 and batch size=50, while molecular fingerprint and CNN use the same batch size, but the model converges in only 15 iterations. The parameter *ncomponents* of PCA is configured to 0.9, which means using a number of components sufficient to consider 90% of variance. Meanwhile, the decision tree algorithm is applied with a threshold of 0.005, indicating that only the features with an importance score greater than 0.005 can be retained. The knn classifier parameter *n* neighbors is set to 3, while random state is 42 for logistic regression, random forest and decision tree to ensure consistency of the classification results over multiple runs.

### Evaluation metrics

The evaluation metrics for model performance are Accuracy, Precision, Recall and F1 score. Classification results have four types: true positive(TP), false negative(FN), false positive(FP) and true negative(TN). Accuracy represents the proportion of all records with correct predictions out of the total. Precision is the percentage of true predictions in all predicted positive results. Recall is the proportion of correctly predicted positive molecules to all actual positive ones. The Precision and Recall rate are interdependent, with one affecting the other. F1 score represents the average of them, with higher values indicating better model quality. These metrics are calculated as follows:

1 $$\begin{aligned} Accuracy= & {} \frac{TP+TN}{TP+TN+FP+FN} \end{aligned}$$

2 $$\begin{aligned} Precision= & {} \frac{TP}{TP+FP} \end{aligned}$$

3 $$\begin{aligned} Recall= & {} \frac{TP}{TP+FN} \end{aligned}$$

4 $$\begin{aligned} F1= & {} \frac{2*Precision*Recall}{Precision+Recall} = \frac{2*TP}{2*TP+FP+FN} \end{aligned}$$

### Performance evaluation on predicting molecular activity

For GPR151 activator activity prediction task, this paper first compares the performance of three raw molecular features (RDKFP, MorganFP, Mol2vec) and five basic classifiers (LR, KNN, RF, DT, SVM) using the test set’s performance metrics as the benchmark result. The results are shown in Table 4. The experiment findings indicate that MorganFP outperforms Mol2vec on logistic regression, decision tree and svm classifiers and doesn’t perform well on knn and random forest. However, MorganFP and Mol2vec outperform RDKFP features overall. The svm classifier achieves best performance on molecular fingerprint features, while knn is the best on Mol2vec features. Moreover, although the accuracy for the three molecular features on five classifiers reach 0.85, F1 scores are all unsatisfactory. The results indicate that the models perform poorly in selecting active molecules.

**Table 4:** Performance comparision of the test set on five traditional classifiers, and the bold marks the best in the group

Feature (raw)	Classifier	Accuracy	Precision	Recall	F1
RDKFP	LR	0.8643	0.6462	0.5638	0.6022
	KNN	0.8658	0.7462	0.3992	0.5201
	RF	0.8651	0.8	0.3457	0.4828
	DT	0.8043	0.4619	0.4486	0.4551
	SVM	**0.8898**	**0.8478**	**0.4815**	**0.6142**
MorganFP	LR	0.8928	0.7404	0.6377	0.6829
	KNN	0.8598	0.8043	0.3045	0.4418
	RF	0.8741	0.8378	0.3827	0.5254
	DT	0.8568	0.6313	0.5144	0.5669
	SVM	**0.9018**	**0.8256**	**0.5844**	**0.6843**
Mol2vec	LR	0.8928	0.766	0.5926	0.6682
	KNN	**0.8921**	**0.7346**	**0.6379**	**0.6828**
	RF	0.8838	0.775	0.5103	0.6154
	DT	0.8313	0.5375	0.5309	0.5342
	SVM	0.8898	0.7727	0.5597	0.6492

To extract and learn better features, we perform the preprocessing operation shown in Fig. 2 for rdk and morgan fingerprint and input them into three network structures of CNN, LSTM and Bi-LSTM. In addition, we extract features by traditional feature selection methods to compare with deep models, then feed them into the same five classifiers to evaluate the efficacy of our feature extraction algorithm. Table 5 shows the performance results of different neural network models and classifiers on RDKFP, while Table 6 presents the results on MorganFP. The trend column in both tables depicts the performance change of the new model compared with benchmark model in Table 4 ($\uparrow$ means better, − means almost the same and $\downarrow$ means worse). Table 8 shows the performance comparison between the proposed algorithm in the paper and the traditional feature selection methods for different molecular features. Among the feature selection methods are PCA [27], LDA [28] and DTA [29]. The table displays the best results obtained by combining traditional methods with five classifiers and the optimal model from Table 5, 6 and 7. It can be seen that our algorithm works better and gains a major performance improvement.

**Fig. 4:**
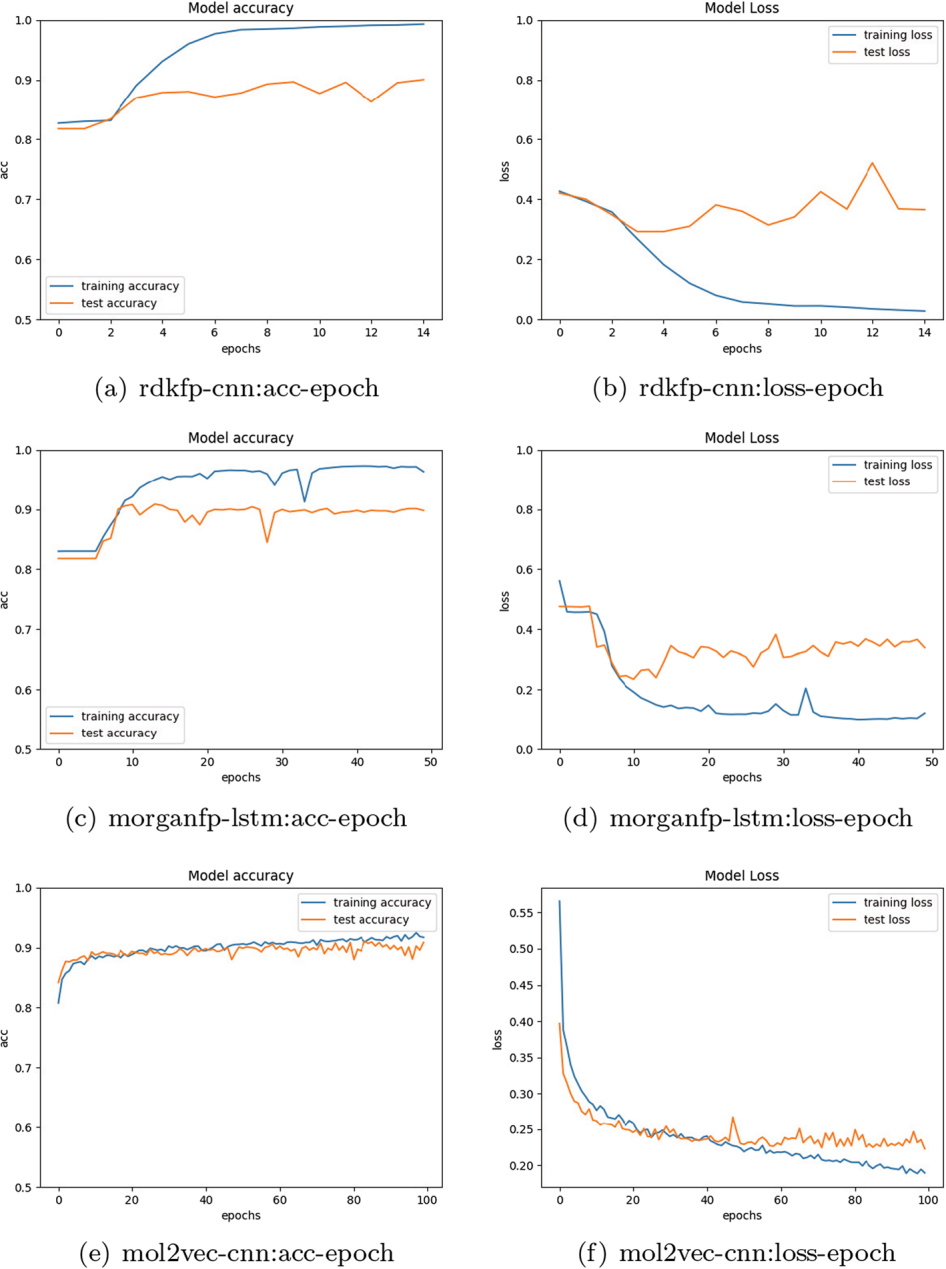
Iteration curves of accuracy and loss values for RDKFP-CNN (epoch=15), MorganFP-LSTM (epoch=50), Mol2vec-CNN (epoch=100) on train and test sets are from top to bottom. As iteration increases, the accuracy of train and test sets rises while loss value decreases in the fluctuation until the convergence

Table 5 demonstrates that after processing and feature extraction using CNN, F1 score of the RDKFP in test set improves by approximately 8%. Furthermore, logistic regression, knn and svm achieve significant improvement compared to the raw RDKFP feature, with knn displaying the best performance. However, random forest and decision tree are not effective. After processing and LSTM/Bi-LSTM feature extraction, RDKFP shows improvement in all five basic classifiers. The poorly model of random forest and decision tree in Table 4 also demonstrates great improvement, with F1 score increasing from 0.5 to approximately 0.62. Table 6 illustrates that MorganFP performs best after processing and LSTM feature extraction, with F1 score increasing by approximately 4% to reach 0.73. The F1 scores of all five classifiers improved compared to the raw MorganFP features. The F1 scores of all classifiers reached 0.7 or higher, except for the decision tree. Table 7 indicates that Mol2vec performs best after CNN feature extraction, with F1 score improving by approximately 5% to reach 0.73. There is an enhancement on all classifiers compared to the raw Mol2vec features, with the svm achieving the accuracy of 0.92 and F1 score of approximately 0.76. Figure 4 presents the accuracy and loss iteration curves of three optimal model RDKFP-CNN, MorganFP-LSTM and Mol2vec-CNN for train and test sets during training process.

### Generalization experiment

Professor John J. Irwin of UCSF [38] released ZINC20 (*zinc*20.*docking*.*org*) in 2020, which contains over 1.4 billion compounds. After consistent collection, we select 300 active and 500 inactive molecules from the AID1508602 dataset as the parent samples. Similar molecules of GPR151 activator are obtained from ZINC20-ForSale-22Q1-1.6B database and further filtered according to the values of ecfp4 and daylight, which are both greater than the value of 0.5. After de-duplication, the final number of molecules is about 10k.

**Fig. 5:**
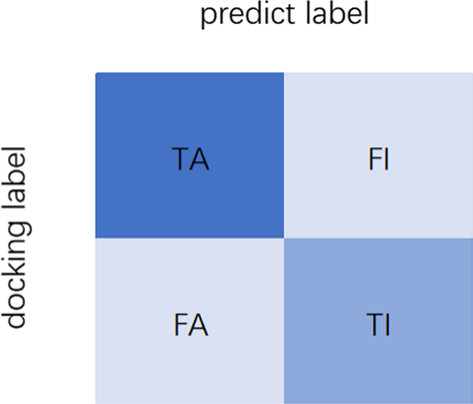
Confusion matrix of docking and predicted labels

To validate the efficacy of the model in this study, we conduct experiments to compare the activity prediction results of the model with the docking computational results in the set of above molecules from above mentioned ZINC database. Molecular docking computation is performed using AutoDock GPU with the same environment and parameter settings as reported in the docking preparation section. The consistency between docking results and the ideal three predicted models (RDKFP-CNN, MorganFP-LSTM, Mol2vec-CNN-SVM) is evaluated through three metrics: Consistency, Active-Recall and Inactive-Recall which can be calculated by the following equations:

5 $$\begin{aligned} Consistency= & {} \frac{TA+TI}{TA+TI+FA+FI} \end{aligned}$$

6 $$\begin{aligned} Active-Recall= & {} \frac{TA}{TA+FI} \end{aligned}$$

7 $$\begin{aligned} Inactive-Recall= & {} \frac{TI}{TI+FA} \end{aligned}$$

where Consistency denotes the overall agreement between docking and prediction results, Active-Recall and Inactive-Recall indicate the recall value of docking active molecules and the recall value of docking inactive molecules, respectively. “A” and “I” represent active and inactive and the confusion matrix is shown in Fig. 5. Table 9 displays the statistical results of consistency evaluation. The best model Mol2vec-CNN-SVM gains a consistency of 71.6%, with a recall rate of 76.1% for the docking active molecules. The results suggest that our model is able to identify most of the docking active molecules. Therefore, Mol2vec-CNN-SVM provides a promising approach for the preliminary screening and enhances the efficiency of virtual screening from massive datasets.

**Fig. 6:**
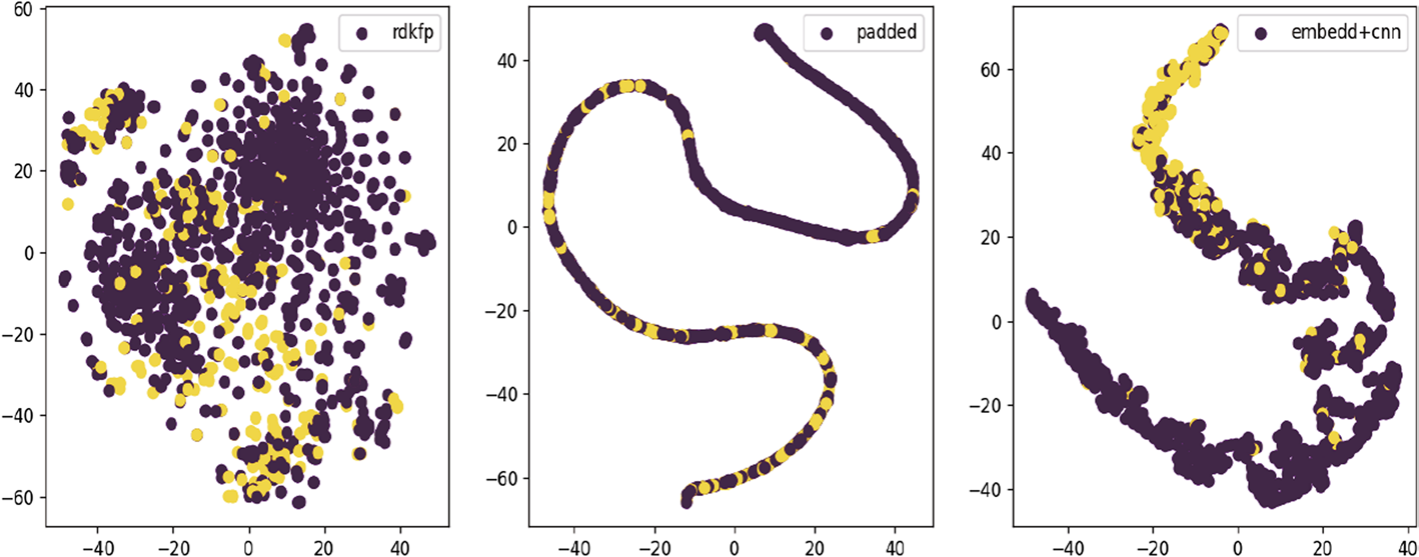
Visualization of rdk fingerprint feature extraction steps on best model CNN. From left to right are the features of raw rdk fingerprint, after encoding and padding, after embedding operation and extracted by CNN

**Fig. 7:**
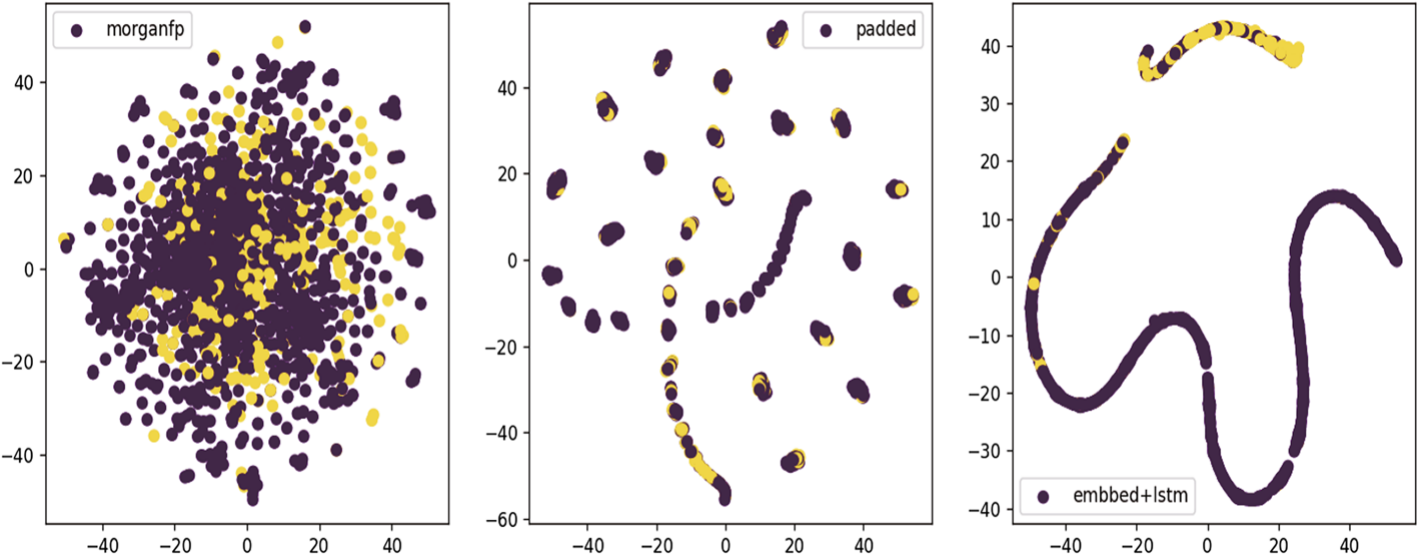
Visualization of morgan fingerprint feature extraction steps on best model LSTM. From left to right are the features of raw morgan fingerprint, after encoding and padding, after embedding operation and extracted by LSTM

**Fig. 8:**
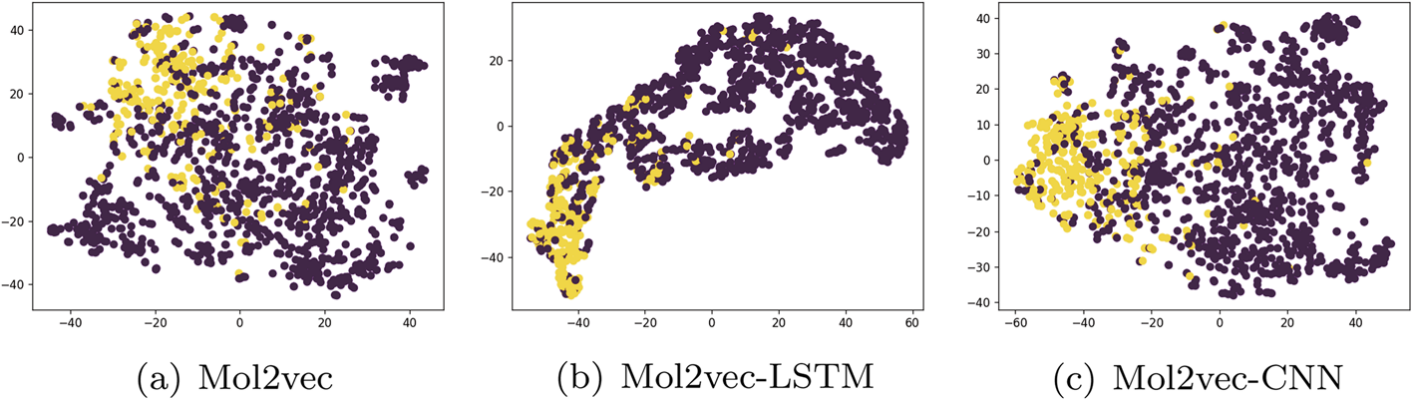
Visualization of Mol2vec feature extraction on LSTM and CNN model

**Table 5:** Evaluation of neural network models and classifiers on test set performance with processed rdk fingerprint, and the bold marks the best in the table

Feature	Classifier	Accuracy	Precision	Recall	F1	Trend
RDKFP processed	CNN	**0.9003**	**0.7806**	**0.6296**	**0.697**	$\uparrow$
	LSTM	0.8748	0.6759	0.6008	0.6362	$\uparrow$
	Bi-LSTM	0.8823	0.724	0.572	0.6391	$\uparrow$
	CNN+LR	0.8913	0.7988	0.5391	0.6437	$\uparrow$
	CNN+KNN	0.8808	0.7121	0.5802	0.6395	$\uparrow$
	CNN+RF	0.8546	0.6992	0.3539	0.4699	$\uparrow$
	CNN+DT	0.8493	0.8088	0.2263	0.3537	$\downarrow$
	CNN+SVM	0.8913	0.7988	0.5391	0.6437	$\downarrow$
	LSTM+LR	0.8748	0.6712	0.6132	0.6409	$\uparrow$
	LSTM+KNN	0.8763	0.6857	0.5926	0.6358	$\uparrow$
	LSTM+RF	0.8718	0.6765	0.5679	0.6174	$\uparrow$
	LSTM+DT	0.8718	0.6765	0.5679	0.6174	$\uparrow$
	LSTM+SVM	0.8748	0.6776	0.5967	0.6346	$\uparrow$
	Bi-LSTM+LR	0.8808	0.7234	0.5597	0.6311	$\uparrow$
	Bi-LSTM+KNN	0.8816	0.7202	0.572	0.6376	$\uparrow$
	Bi-LSTM+RF	0.8756	0.6935	0.5679	0.6244	$\uparrow$
	Bi-LSTM+DT	0.8711	0.6802	0.5514	0.6091	$\uparrow$
	Bi-LSTM+SVM	0.8801	0.7044	0.5885	0.6413	$\uparrow$

**Table 6:** Evaluation of neural network models and classifiers on test set performance with processed morgan fingerprints, and the bold marks the best in the table

Feature	Classifier	Accuracy	Precision	Recall	F1	Trend
MorganFP processed	CNN	0.8898	0.7124	0.6625	0.6866	-
	LSTM	**0.8981**	**0.7082**	**0.749**	**0.728**	$\uparrow$
	Bi-LSTM	0.8793	0.683	0.6296	0.6552	$\downarrow$
	CNN+LR	0.8883	0.7238	0.6255	0.6711	$\downarrow$
	CNN+KNN	0.8756	0.6954	0.5638	0.6227	$\uparrow$
	CNN+RF	0.8853	0.7368	0.5761	0.6467	$\uparrow$
	CNN+DT	0.8748	0.7639	0.4527	0.5685	-
	CNN+SVM	0.8906	0.7389	0.6173	0.6726	$\downarrow$
	LSTM+LR	0.8973	0.707	0.7449	0.7255	$\uparrow$
	LSTM+KNN	0.8958	0.7114	0.7202	0.7157	$\uparrow$
	LSTM+RF	0.8921	0.6926	0.7325	0.712	$\uparrow$
	LSTM+DT	0.8831	0.6835	0.6667	0.675	$\uparrow$
	LSTM+SVM	0.8973	0.707	0.7449	0.7255	$\uparrow$
	Bi-LSTM+LR	0.8748	0.6667	0.6255	0.6454	$\downarrow$
	Bi-LSTM+KNN	0.8763	0.6639	0.6502	0.657	$\uparrow$
	Bi-LSTM+RF	0.8763	0.6639	0.6502	0.657	$\uparrow$
	Bi-LSTM+DT	0.8778	0.677	0.6296	0.6525	$\uparrow$
	Bi-LSTM+SVM	0.8741	0.6623	0.6296	0.6456	$\downarrow$

**Table 7:** Comparison of neural network models and classifiers in Mol2vec test set performance, and the bold marks the best in the table

Feature	Classifier	Accuracy	Precision	Recall	F1	Trend
Mol2vec	CNN	0.9085	0.7895	0.679	0.7301	$\uparrow$
	LSTM	0.8996	0.7444	0.6831	0.7124	$\uparrow$
	Bi-LSTM	0.8988	0.7389	0.6872	0.7122	$\uparrow$
	CNN+LR	0.904	0.7626	0.6872	0.7229	$\uparrow$
	CNN+KNN	0.8958	0.7524	0.639	0.6904	$\uparrow$
	CNN+RF	0.91	0.7971	0.679	0.7333	$\uparrow$
	CNN+DT	0.8718	0.6651	0.5967	0.6291	$\uparrow$
	CNN+SVM	**0.9153**	**0.7928**	**0.7243**	**0.757**	$\uparrow$
	LSTM+LR	0.8973	0.7431	0.6667	0.7028	$\uparrow$
	LSTM+KNN	0.8861	0.7136	0.6255	0.6667	$\downarrow$
	LSTM+RF	0.8973	0.7409	0.6708	0.7041	$\uparrow$
	LSTM+DT	0.8628	0.65	0.535	0.5869	$\uparrow$
	LSTM+SVM	0.8951	0.7191	0.6955	0.7071	$\uparrow$
	Bi-LSTM+LR	0.8973	0.7409	0.6708	0.7041	$\uparrow$
	Bi-LSTM+KNN	0.8921	0.7302	0.6461	0.6856	-
	Bi-LSTM+RF	0.8973	0.7477	0.6584	0.7002	$\uparrow$
	Bi-LSTM+DT	0.8711	0.6621	0.5967	0.6277	$\uparrow$
	Bi-LSTM+SVM	0.9003	0.7523	0.6749	0.7115	$\uparrow$

**Table 8:** Comparison of different feature selection algorithms on test set performance. The results of traditional methods in the table are the best with five classifiers, and the bold marks the best in the group

Feature	Algorithm	Accuracy	Precisionn	Recall	F1
RDKFP	PCA	0.8958	0.8291	0.5391	0.6534
	LDA	0.8583	0.6089	0.6214	0.6151
	DTA	0.8576	0.6626	0.4444	0.532
	CNN	**0.9003**	**0.7806**	**0.6296**	**0.697**
MorganFP	PCA	0.8988	0.7647	0.642	0.698
	LDA	0.8726	0.654	0.6379	0.6458
	DTA	0.8816	0.7707	0.4979	0.605
	LSTM	**0.8981**	**0.7082**	**0.749**	**0.728**
Mol2vec	PCA	0.8816	0.7157	0.5802	0.6409
	LDA	0.8876	0.736	0.5967	0.6591
	DTA	0.8928	0.7525	0.6132	0.6757
	CNN	**0.9153**	**0.7928**	**0.7243**	**0.757**

**Table 9:** The consistency results of molecular docking calculation and model prediction, and the bold marks the best in the table

	Consistency (%)	Active-recall (%)	Inactive-recall (%)
DOCKING:RDKFP-CNN	69	74.5	61
DOCKING:MorganFP-LSTM	70.6	76.1	61.7
DOCKING:Mol2vec-CNN-SVM	**71.6**	**76.1**	**64.8**

## Discussion

As shown in previous results, traditional feature selection algorithms have shown certain advantages compared to initial molecular features. On the other hand, deep learning algorithms exhibits strong learning abilities in high-dimensional features and performs well in the molecular field. From the results section, it appears that the deep model are valid and applicable on different types of molecular features. The encoding and padding operations of molecular fingerprints make the features more dense and lay the foundation for feature input of neural networks. Mol2vec calculates the molecular substructure vector and leverages neural networks to extract highly effective features that can enhance prediction accuracy.

To enhance the interpretability of the model, we attempt to use t-SNE dimensionality reduction and visualization methods. By employing t-SNE, we can effectively visualize the high-dimensional data in a lower-dimensional space, making it easier to explore the correlations and distributions of the features. The feature extraction steps on RDKFP, MorganFP are visualized as shown in Figs. 6 and 7. Figure 8 illustrates the impact of two deep models on the extraction of Mol2vec features. We utilize a scatter plot to visualize the molecular features, where the red dots represent inactive molecules, and yellow dots represent active ones. Effective feature extraction resulted in a clearer separation of the two classes of data, enabling the classifier to determine the activator label more accurately. This is the major reason for the performance improvement. RDKFP+CNN graph has more overlap between two types of data, resulting in a lower F1 score than MorganFP+LSTM. In addition, Mol2vec features also have clear boundaries between two types of data after LSTM and CNN feature extraction. This distribution is appropriate for traditional classifiers, resulting in a significant impact on various classifiers.

The time complexity of the proposed method primarily involves two tasks: calculating molecular features and training the model. While the calculation time for molecular fingerprints and Mol2vec increases linearly with the number of input molecules, the input features of deep neural networks remain constant, resulting in consistent calculation time during model training. The spatial complexity of our method is independent of the number of input molecules, as the dimensions of the molecular fingerprint and Mol2vec features remain fixed at 2048 and 100, respectively.

However, our model still face several challenges. Figures 4b and d show that the loss value of the test set is higher than that of the training set. This suggests to some extent that there may be over-fitting present in the data. To mitigate this issue, we adjust the learning rate and batch size, add Dropout layers, and stop the training process when iterative convergence. Despite our efforts to mitigate over-fitting, it could not be fully eliminated. The characteristics of the GPR151 activator dataset may account for this observation. The different distributions of molecular structures between the train and test sets present a challenge for the learning algorithm. Furthermore, our encoding rules primarily reflect the location of effective bits in molecular fingerprints and are not based on domain-specific expertise. This may further worsen the distribution gap between the molecular features of the train and test sets, potentially leading to over-fitting to some extent. We believe that our algorithm can be further improved by using more domain-specific knowledge to encode molecular fingerprints. Along with the issue of over-fitting, lack of interpretability is also a drawback of neural networks. Visualization techniques are obviously lack of adequate theoretical support. We plan to utilize interpretative machine learning methods to gain a deeper understanding of neural network performance in the future work.

To summarize, the prediction performance of the test set can generally be improved by implementing molecular feature representations after neural network feature extraction. The LSTM model is more suitable for molecular fingerprints, while the CNN model is more appropriate for Mol2vec features. The performance of different classifiers varies, with svm yielding the best result for Mol2vec features extracted by CNN. This model can be applied to screen active molecules from massive databases. Our designed GPR151 activator classification model achieves over 70% accuracy in screening active molecules in large molecular datasets. Furthermore, deep learning model significantly accelerates the screening speed, thereby reducing time consumption. The molecular activity prediction model is executed on a single CPU of 12th Gen Intel(R) Core(TM) i7-12700 while AutoDock Vina and AutoDock GPU are executed on the “ORISE” supercomputers, with calculation time of 109 h on 32 CPUs and 1 h on 4 GPUs to process 10,000 molecules, respectively. However, the prediction time of our deep learning model is controlled within a few minutes, which is ten times faster than traditional molecular docking. This provides a novel method for rapidly enriching potential active compounds for large-scale virtual screening in drug discovery.

## Conclusion

In this paper, we propose a molecular fingerprint enhancement algorithm that preprocesses bit-sparse fingerprint features using the idea of bag-of-words model in natural language processing. By combining this encoding step with neural network models for feature extraction, we can better extract effective information from molecules than traditional feature selection algorithms such as PCA, resulting in improved classifier performance. Moreover, we perform experiments on different types of molecular features, neural networks, and classifiers to systematically compare the adaptability of multiple network structures and classifiers for molecular features. Our optimal model, Mol2vec-CNN-SVM, achieves an accuracy and F1 score of 92% and 76%, respectively. Our model’s effectiveness is demonstrated through generalization experiments on large-scale databases. The model is capable of narrowing down the range of potential compounds in the initial stages of virtual screening through activity classification, which assists molecular docking in rapidly identifying active molecules. In our future research, we will focus on improving the encoding rules and exploring state-of-art artificial intelligence architectures to optimize our proposed method. We will continue to explore molecular property prediction model to provide more reliable results for virtual screening.

## Data Availability

Raw data of GPR151 activators are available in [https://pubchem.ncbi.nlm.nih.gov/bioassay/1508602]. The datasets generated during the current study and code are all available in [https://github.com/xuhuangchao/gpr151_activity_prediction.git]
